# Microbial Populations in Naked Neck Chicken Ceca Raised on Pasture Flock Fed with Commercial Yeast Cell Wall Prebiotics via an Illumina MiSeq Platform

**DOI:** 10.1371/journal.pone.0151944

**Published:** 2016-03-18

**Authors:** Si Hong Park, Sang In Lee, Steven C. Ricke

**Affiliations:** 1 Center for Food Safety, Department of Food Science, University of Arkansas, Fayetteville, AR 72704, United States of America; 2 Cellular and Molecular Biology Graduate Program, University of Arkansas, Fayetteville, AR 72701, United States of America; University of Illinois at Urbana-Champaign, UNITED STATES

## Abstract

Prebiotics are non-digestible carbohydrate dietary supplements that selectively stimulate the growth of one or more beneficial bacteria in the gastrointestinal tract of the host. These bacteria can inhibit colonization of pathogenic bacteria by producing antimicrobial substances such as short chain fatty acids (SCFAs) and competing for niches with pathogens within the gut. Pasture flock chickens are generally raised outdoors with fresh grass, sunlight and air, which represents different environmental growth conditions compared to conventionally raised chickens. The purpose of this study was to evaluate the difference in microbial populations from naked neck chicken ceca fed with commercial prebiotics derived from brewer’s yeast cell wall via an Illumina MiSeq platform. A total of 147 day-of-hatch naked neck chickens were distributed into 3 groups consisted of 1) C: control (no prebiotic), 2) T1: Biolex^®^ MB40 with 0.2%, and 3) T2: Leiber^®^ ExCel with 0.2%, consistently supplemented prebiotics during the experimental period. At 8 weeks, a total of 15 birds from each group were randomly selected and ceca removed for DNA extraction. The Illumina Miseq platform based on V4 region of 16S rRNA gene was applied for microbiome analysis. Both treatments exhibited limited impact on the microbial populations at the phylum level, with no significant differences in the OTU number of *Bacteroidetes* among groups and an increase of *Proteobacteria* OTUs for the T1 (Biolex^®^ MB40) group. In addition there was a significant increase of genus *Faecalibacterium* OTU, phylum *Firmicutes*. According to the development of next generation sequencing (NGS), microbiome analysis based on 16S rRNA gene proved to be informative on the prebiotic impact on poultry gut microbiota in pasture-raised naked neck birds.

## Introduction

The differences in broiler chicken growth systems between conventional environmentally controlled housing and on pasture include rearing environments, feed components and what are considered acceptable dietary additives. Broiler chickens raised in conventional rearing systems are supplemented with antimicrobial agents or growth promoters for the purposes of production enhancement as well as improved health attributes. However, pasture raised chickens, as an alternative bird rearing system, are grown on fresh grass, outdoor environments without most traditional growth promoters except for feed additives considered natural or organic such as prebiotics [1–3]. Conventional commercial breeds are not always suitable for the longer growth periods and environmental conditions prevalent for outdoor management systems. Although slow-growing broiler breeds such as naked neck chicken, require a longer growth period (as much as 12 weeks) compared to fast-growing one (7 weeks), slow-growing broiler do yield a better gait score, improved livability and nutritional differences in meat quality [4, 5].

However, issues such as occurrence of foodborne pathogens and diminished health status can be a chronic problem in some of the nonconventional systems [6]. Even in more conventional poultry production systems, emergence of multidrug resistant bacteria in poultry products and rearing system environments as well as the demands for high quality foods by consumers has accelerated the development of alternative feed amendments [2, 6]. Functional feed additives such as prebiotics have been examined in the past several decades as potential dietary additives to limit pathogenic bacteria establishment in humans and improve gut health in poultry and other food animals [7–10].

Prebiotics are not digestible by the host but commensal bacteria in the gut can metabolize them to produce short chain fatty acids (SCFAs) [8] and in some cases bacteriocins to inhibit the colonization of pathogenic bacteria in the gut as well as select for beneficial bacteria such as *Lactobacillus* and *Bifidobacteria*. Furthermore, since the complex ecosystem of chicken gastrointestinal tracts (GIT) plays an important role in nutrient utilization [11], growth development [12], detoxification [13], villi and crypt promotion [11], it is crucial to maintain microbial populations that support healthy host conditions [14]. Several commercial prebiotic type components have been generated from yeast cells including cell walls and fermentation products [15–17]. These prebiotic type components not only have positive effects on animal and poultry productivity but also contribute to a healthy gut physiology along with the increased shift towards beneficial microorganisms [15–18].

Since prebiotics have a potential impact on gut health and are generally recognized as safe (GRAS) status, nonconventional food animal production systems can potentially use prebiotics to enhance productivity efficiency. While several reports have evaluated the effects of prebiotics on growth performance and meat quality [4, 9, 10, 19], the impacts of prebiotics on gut microbiota in nonconventional food animal production systems have not been fully explored [2]. Although a denaturing gradient gel electrophoresis (DGGE) technique has been widely utilized to investigate the microbiota differences between control and treatments, similar banding patterns on a gel make it difficult to interpret differences as well as the problems associated with the low DNA recovery rate from the gel for sequencing being imprecise for delineating the more subtle changes in microbiota [2, 20, 21].

High-throughput next generation sequencing (NGS) platforms based on 16S rRNA gene amplicons can serve as a phylogenetic markers to explore the more complex aspects of the microbiome in humans and animals [12, 22–26]. Amplicon sequencing using an Illumina MiSeq platform is rapidly increasing since the MiSeq possesses a lower cost per sequence compared to other platforms [27] and generates 7.5 Gb from 15 million 250-base paired-end reads within 3 days as well as longer sequences when using the MiSeq Reagent Kit v2 500 cycles (Illumina, San Diego, CA, USA).

Even though there have been several trials involving the naked neck breed of birds in pasture flocks, most of them have focused on the reduction of pathogens such as *Salmonella* and *Campylobacter* and growth performance [4, 9, 21, 28]. Investigation of the gut microbiota in naked neck chickens raised on pasture flock was initiated in the current study to focus on the implications of prebiotic roles on commensal microorganisms as a follow-up to an earlier study [21]. To the best of our knowledge, this is the first study to investigate pasture flock raised naked neck chicken cecal microbiota fed with yeast-based prebiotics using the Illumina MiSeq platform. In order to evaluate the effects of two commercial prebiotics Biolex^®^ MB40 and Leiber^®^ ExCel derived from yeast cell walls on chicken gut microbiota, naked neck chickens were grown up to 8 weeks on pasture flock fed with one of these prebiotics. Total DNA isolated from each cecum were utilized for sequencing using the Illumina MiSeq platform and quantitative insights into microbial ecology (QIIME) pipeline as a bioinformatics tool was adopted for sequencing data analysis and interpretation.

## Materials and Methods

### Chicken housing

A total of 147 naked neck chicks (Peterson Farms, Decatur, AR, USA) were randomly allocated to 3 pens (49 birds per each pen) with feed and water *ad libitum* for the duration of the 8 week experimental period. The birds and each pen were relocated within the pasture twice a week to supply fresh growing conditions. One pen served as the control (C) group fed with only genetically modified organism (GMO)-free feeds (Hiland Naturals, Killbuck, OH, USA) and the other two groups (T1 and T2) were fed with one of the respective yeast-based prebiotics with typical feed mixtures in the starter, grower and finisher rations. The feeds of both T1 and T2 groups were mixed with prebiotics Biolex^®^ MB40 (0.2%, Leiber GmbH, Hafenstraße, Germany) derived from yeast cell (*Saccharomyces cerevisiae*) walls included beta-D-glucan and mannan-oligosaccharides (MOS) and Leiber^®^ ExCel (0.2%, Leiber GmbH) which is similar to Biolex^®^ MB40, respectively, and supplemented for the duration of the experimental period. The Institute Animal Care and Use Committee (IACUC) approval was exempted for this research because all birds were commercially raised in an off-campus facility and limited to microbiological evaluation.

### Sample collection

At 8 weeks, a total of 45 birds (15 birds per group) were randomly chosen and humanely euthanized with CO_2_ gas. The extracted ceca were immediately transferred to sterile Whirl-Pak^®^ bags (Nasco, Fort Atkinson, WI, USA) individually and stored -20°C until DNA extraction. The remainder of the birds were processed and the respective performance responses were described previously [21].

### DNA extraction

A total of 200 mg of cecal contents from each bird was utilized for DNA isolation using a QIAamp DNA Stool Mini Kit (Qiagen, Valencia, CA, USA) with modifications to increase DNA concentration. The specific DNA extraction protocol was described previously [21]. Isolated DNA concentration was measured using a Qubit^®^ 2.0 Fluorometer (Life Technology, Carlsbad, CA, USA) and diluted to 10 ng/μL.

### Library preparation

A 10 ng of DNA aliquot isolated from each cecal content sample was utilized to construct a sequencing library targeting the V4 region of 16S rRNA following a previous report [29]. For library preparation, 40 samples from another independent study were combined with 45 samples to simultaneously sequence both sets of samples. In brief, individual DNA samples were amplified with dual-index primers via PCR and normalized amplicons using a SequalPrep^™^ Normalization kit (Life Technology) according to the manufacturer’s recommendation. Each sample possessed specific barcode sequences at the front and end of the PCR amplicon to discriminate among each other in the pooled library. A five microliter aliquot of each normalized sample was combined to generate 1 pooled library for further assays. Both library concentration and an exact product size were measured using a KAPA Library Quantification Kit (Kapa Biosystems, Woburn, MA, USA) through a quantitative PCR (qPCR, Eppendorf, Westbury, NY, USA) assay and an Agilent 2100 Bioanalyzer System (Agilent, Santa Clara, CA, USA), respectively. Based on the qPCR and bioanalyzer results, the pooled library was subsequently diluted to 4 nM prior to sequencing.

### Sequencing via an Illumina MiSeq platform

A pooled library (20 nM) and a PhiX control v3 (20 nM) (Illumina) were mixed with 0.2 N fresh NaOH and HT1 buffer (Illumina) to produce the final concentration at 12 pM each. The resulting library was mixed with the PhiX control v3 (5%, v/v) (Illumina) and 600 uL loaded on a MiSeq^®^ v2 (500 cycle) Reagent cartridge for sequencing. All sequencing procedures were monitored through the Illumina BaseSpace^®^ website.

### Sequencing data processing

Both demultiplexed R1 and R2 sequencing reads (approximately 250 bp in length) files were acquired from the Illumina BaseSpace^®^ website and data processing were performed using a QIIME pipeline (version 1.9.0) [30]. The clustered sequences were utilized to construct Operational Taxonomic Units (OTUs) tables with 97% identity and representative sequences were classified into the respective taxonomical level from phylum to genus based on the Greengenes 16S rRNA gene database. Subsequently alpha diversity (rarefaction curve for OTUs, Chao1, and PD_Whole_Tree) and beta diversity using weighted and unweighted UniFrac distance among samples were generated within the QIIME 1.9.0 package.

## Results and Discussion

### Gastrointestinal tract microbiota analysis

The chicken ceca, as a fermentation chamber, not only play important roles such as polysaccharide digestion, water adsorption and urea recycling but also have the greatest gastrointestinal microbial populations that include an abundance of phylogenetic groups such as *Clostridiales* and *Bacteroidetes* [31, 32]. Recently, Sergeant et al. [26] reported that chicken ceca possess approximately 700 bacterial species based on 16S rRNA amplicon pyrosequencing.

In order to investigate chicken gut microbial populations, several approaches based on viable cells via cultural methods and molecular technologies such as DGGE have been adopted and utilized [21, 33, 34]. The DGGE based on DNA extracted from chicken cecal contents has been utilized as an alternative molecular-based technology over culture-dependent methods; however it does not necessarily represent overall microbiota due to limitations such as low bacterial discernment and insufficient diversity representation [20, 21, 35, 36, 37]. As an alternative approach, the qPCR assay based on 16S rRNA gene clones has been utilized to quantify complex microbiota in chicken ceca [38, 39] but the qPCR assay also has limitations such as PCR primers bias [40].

The NGS technology has been developed over past decade and decreasing costs per sequence has allowed for enhanced characterization and profiling of microbiota in complex ecosystems [27]. The Illumina MiSeq platform, bench top sequencer, based on 16S rRNA amplicons is widely utilized to generate 1.5 Gb per one day with 5 million 150-base paired-end reads [27]. Zhao et al. [41] evaluated the influence of different genotypes and gender on the corresponding chicken fecal gut microbiome based on the V4 regions of 16S rRNA using the Illumina MiSeq platform. A total of 68 out of 190 microbiome species were affected by gender and genotypes and 16 species were identified as *Lactobacillus*. In addition, Fadrosh et al. [22] utilized a similar dual-indexing approach used in the current study for multiplexed 16S rRNA amplicon sequencing based on the Illumna MiSeq platform.

### Sequencing data analysis with QIIME

The Illumina MiSeq was performed using 45 independent samples to generate a total 11,413,205 raw sequence reads (Table 1). After passing the quality filter in an Illumina BaseSpace^®^, 10,179,674 (89.1%) an average of 236,737 reads (Table 1) were utilized for downstream analysis via the QIIME (1.9.0) pipeline [30]. The range of reads used in QIIME analysis was 89,350 to 281,996 and the information for each of the sample reads are illustrated in the S1 Table.

**Table 1:** Sequencing data analysis.

Total reads	PF reads*	Reads identified (PF)	Error rate	% ≥ Q30
11,413,205	10,179,674	89.1%	1.35%	80.6

*PF reads: Reads number after passing filter (PF).

Sequencing data in this study exhibited a 1.35% error rate and 80.6% quality score, respectively (Table 1). Error rate is considered an important factor to evaluate the sequencing quality and associated with quality score (% ≥ Q30) reported by the instrument [29]. A low quality score can increase the error rate including nucleotide substitutions, insertions, deletions, and ambiguous base calls [29].

### Microbial correlation among groups

**Fig 1:**
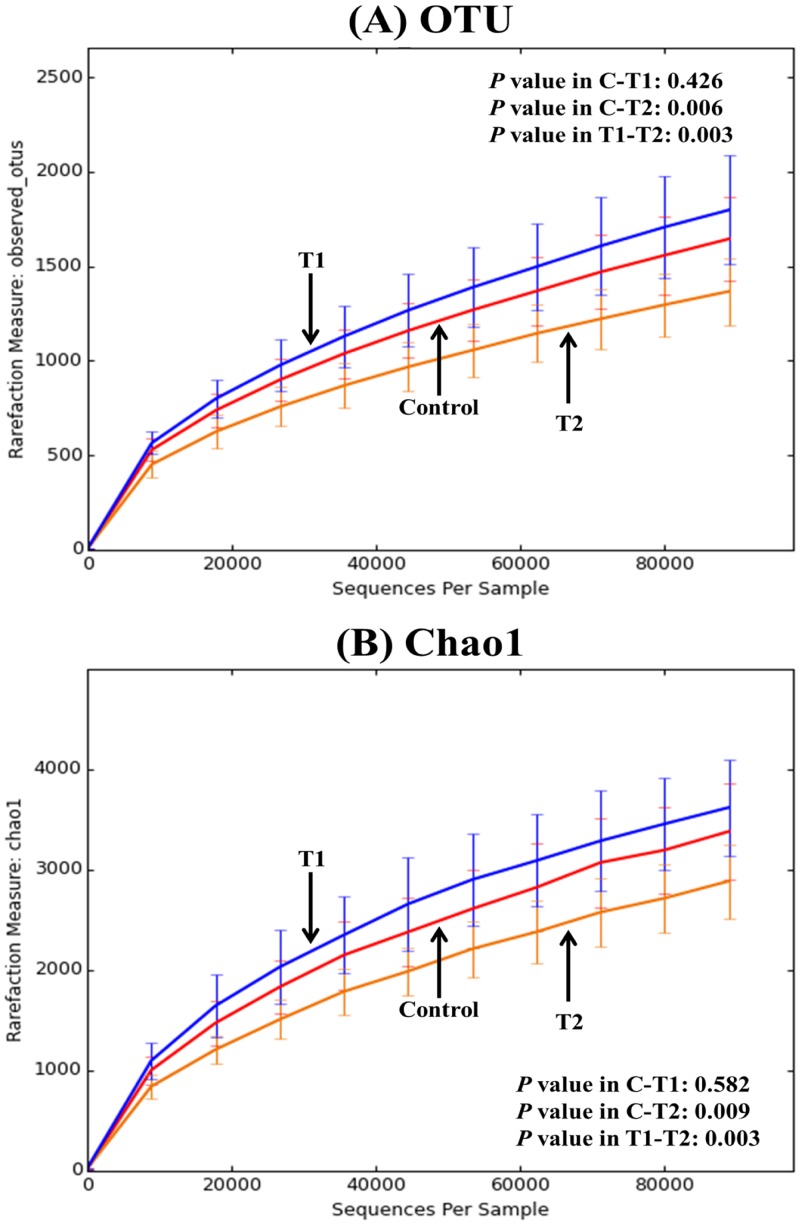
Rarefaction curves of alpha diversity among groups. **(**A) average observed_OTUs at 97% of similarity and (B) average Chao1; Control: normal feed, T1: normal feed with 0.2% Biolex^®^ MB40 and T2: normal feed with 0.2% Leiber^®^ ExCel.

Both alpha and beta diversity were generated using the QIIME 1.9.0 package with a script core_diversity_analyses.py. For alpha diversity analysis, each rarefaction of average observed_OTUs and Chao1 per group is shown in Fig 1A and 1B, respectively. As shown in Fig 1A, T1 (Biolex^®^ MB40) and control groups possessed similar unique OTU numbers, while T2 (Leiber^®^ ExCel) exhibited a significantly lower specific OTU number compared to the other two groups. The Chao1 rarefaction plot (Fig 1B), estimating species richness, also displayed similar patterns to the observed_OTU rarefaction.

**Fig 2:**
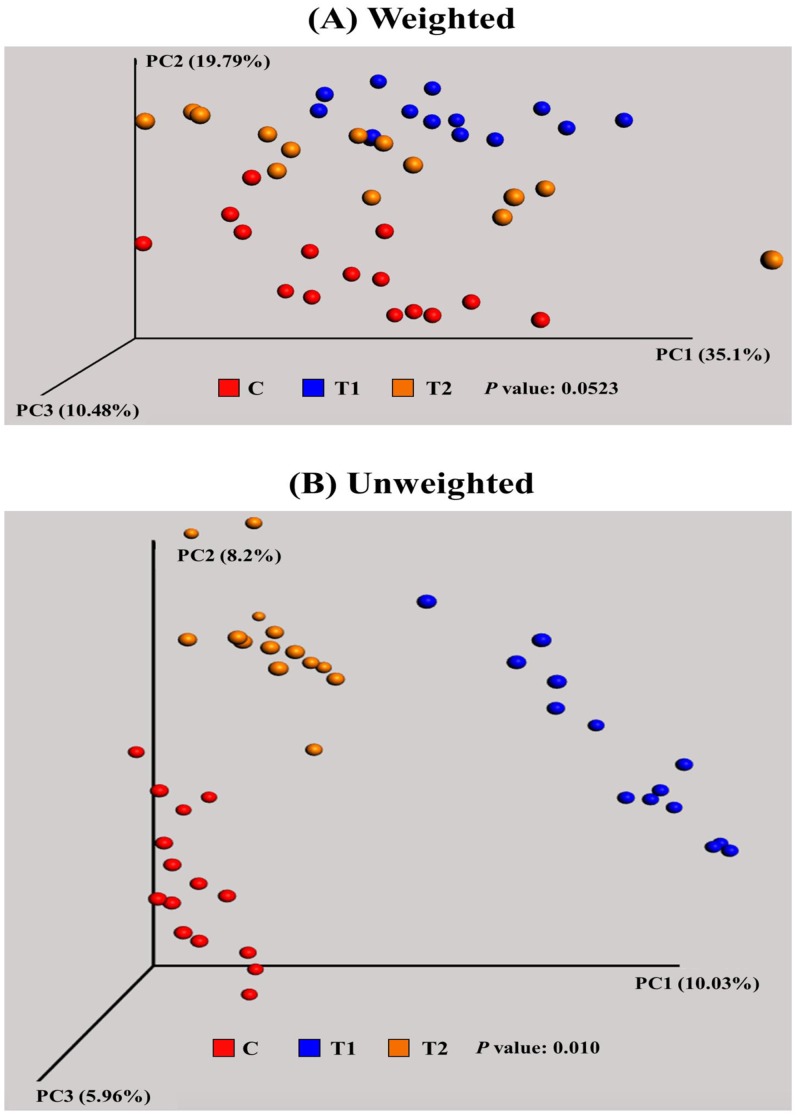
Beta diversity analysis among groups. (A) weighted and (B) unweighted UniFrac PCoA plots of individual birds in each group. Individual sample was represented as spot with red (C; normal feed), blue (T1; normal feed with 0.2% Biolex^®^ MB40), and orange (T2; normal feed with 0.2% Leiber^®^ ExCel).

In the beta diversity analysis, both weighted and unweighted principal coordinated analysis (PCoA) UniFrac plots were generated using a total of 43 samples (Fig 2A and 2B). The PCoA plot served as a multivariate statistical method to indicate the phylogenetic distance between samples with 2 or 3 dimensional presentation diagrams. The weighted PCoA UniFrac plot exhibited the relative abundance of OTUs among group, while the unweighted PCoA UniFrac plot represented the phylogenetic distance based on the presence/absence of OTUs among samples. Fig 2A illustrates the weighted PCoA UniFrac plot and each dot (individual sample) in each group aligned in parallel on the PC1 (41.23%) axis, while each group is clustered distinctively in the unweighted PCoA UniFrac plot as shown in Fig 2B.

### Taxonomic summary

**Fig 3:**
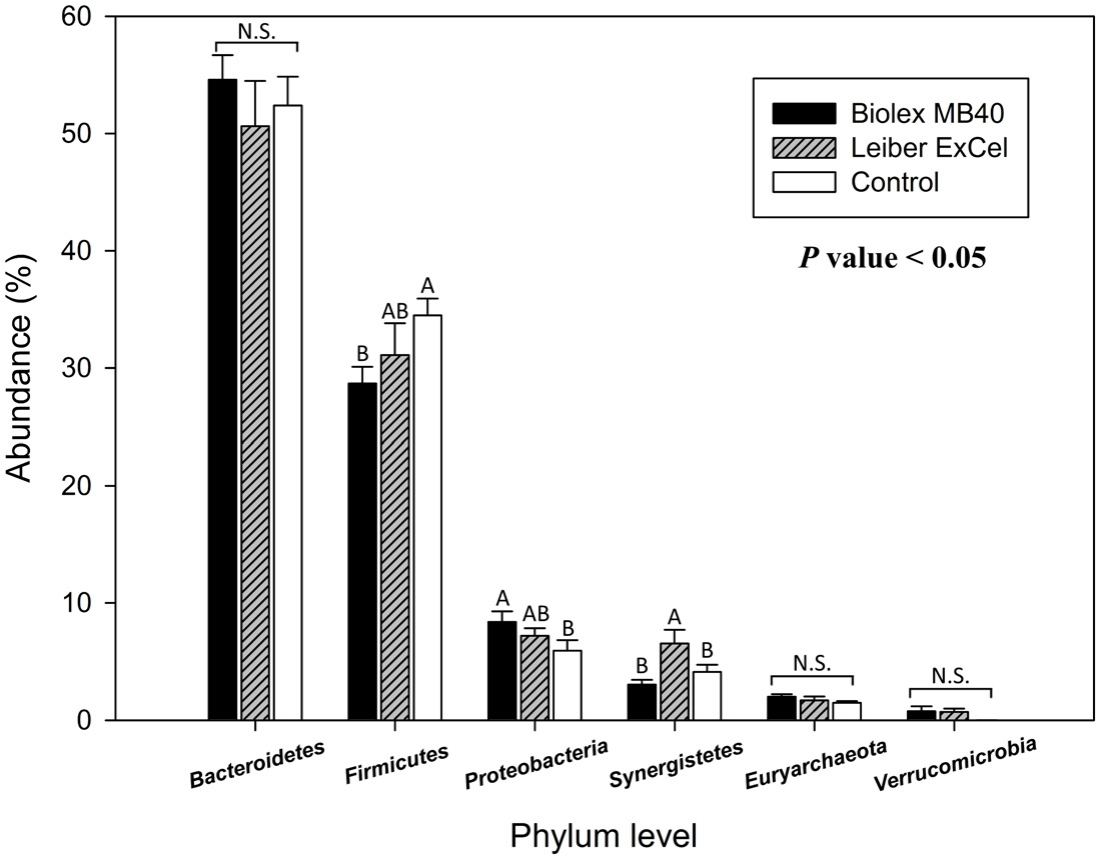
Comparison of top 6 cecal bacteria among groups in a phylum level. Blue, red, and green bars stand for T1 (normal feed with 0.2% Biolex^®^ MB40), T2 (normal feed with 0.2% Leiber^®^ ExCel), and control (C; normal feed), respectively. Capital letters on the top of bar within each phylum level indicate significant differences among groups (*P* < 0.05). N.S. (not significant).

**Fig 4:**
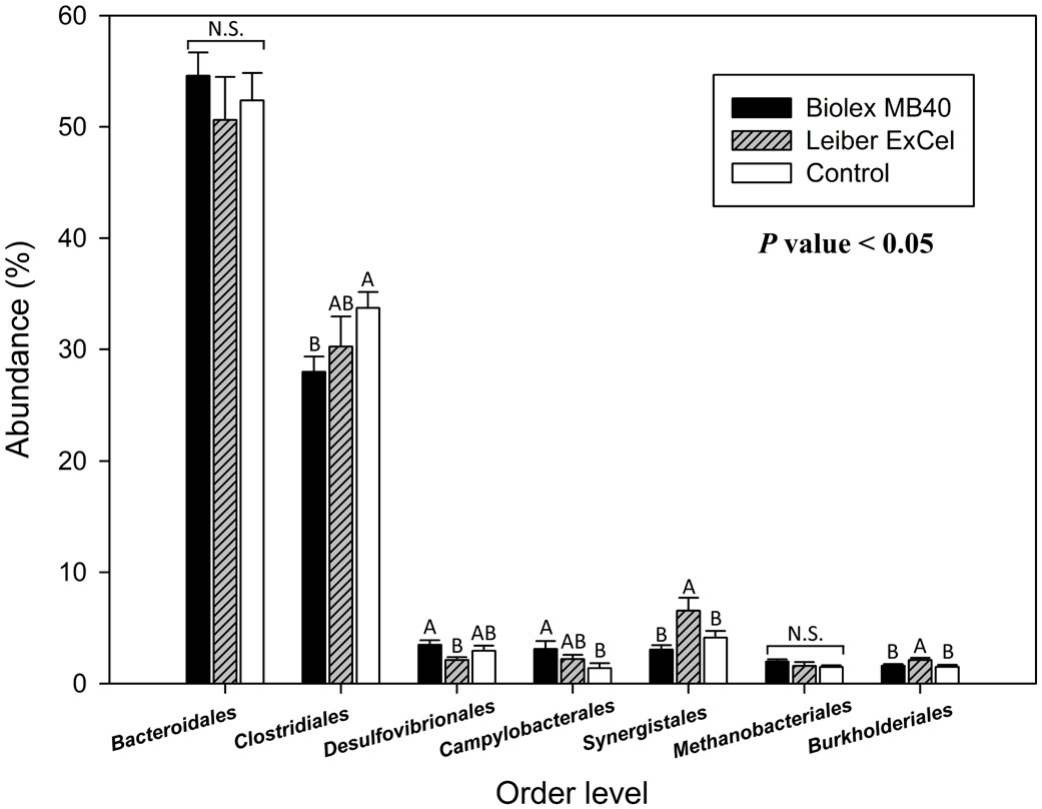
Comparison of top 7 cecal bacteria among groups in an order level. Blue, red, and green bars stand for T1 (normal feed with 0.2% Biolex^®^ MB40), T2 (normal feed with 0.2% Leiber^®^ ExCel), and control (C; normal feed), respectively. Capital letters on the top of bar within each order level indicate significant differences among groups (*P* < 0.05). N.S. (not significant).

All bacterial group taxonomical summaries from phylum to genus levels in individual cecal contents and relative OTUs abundances are illustrated in S1 Fig and S2 Table. Both Figs 3 and 4 represent the top 6 and 7 bacterial groups at a phylum and order level, respectively. In the Fig 3, there were no apparent differences in *Bacteroidetes* and *Euryarchaeota* among groups, while the control group exhibited a significantly greater OTU abundance for *Firmicutes* (Fig 3). The T1 (Biolex^®^ MB40) group revealed relatively high OTUs abundances in *Proteobacteria* and *Cyanobacteria* compared to the other two groups (T2; Leiber^®^ ExCel and control) and *Synergistetes* OTUs was the highest in T2 (Leiber^®^ ExCel) group.

According to the previous reports by Ley et al. [42] and Mariat et al. [43], a decrease in the phylum *Firmicutes*/*Bacteroidetes* ratio is directly associated with a weight loss in humans and mice. In this study, there was no significant difference in the *Firmicutes*/*Bacteroidetes* ratio among groups (*P* > 0.05, data not shown) and this result is highly consistent with a previous report on these birds [21] which included the body weight responses of these same birds which served as the source for the cecal DNA samples used for sequencing in the current study. Although microbiota in chicken ceca evolves from birth to death, several papers have reported that *Firmicutes* remain the predominant bacteria in bird ceca raised in conventional rearing systems [12, 44–46]. However, *Bacteroidetes* do appear to be the predominant bacteria in chickens raised on pasture perhaps due to the different rearing systems [47], bird type [45] and dietary diversity dependent on exposure to sources such as consistent consumption of insects outdoors.

In the top 7 bacterial groups at the order level, *Bacteroidales* and archaeal *Methanobacteriales* were found in chicken ceca [48], but both bacteria were not significantly different among the treatment groups in the current study (Fig 4). The control group did reveal a significant level of OTUs corresponding to *Clostridiales*, while the other three bacterial groups (*Campylobacterales*, *Synergistales* and *Burkholderiales*) exhibited lower OTUs abundances in the control group. Danzeisen et al. [45] compared the chicken cecal microbiome fed with antibiotics which have historically been used in the U.S. poultry industry [49] in order to demonstrate microbial changes impacted by age and treatments. Danzeisen et al. [45] also concluded *Clostridiales* is a predominant order in the *Firmicutes* phylum. In addition, Lu et al. [50] when evaluating the bacterial communities in ceca from mature broilers over time up to 49 days via 16S rDNA clone library reported that *Clostridiaceae* were detected as a predominant group in samples from all ages of birds.

### Bacterial OTUs abundances in family and genus taxonomic level

**Fig 5:**
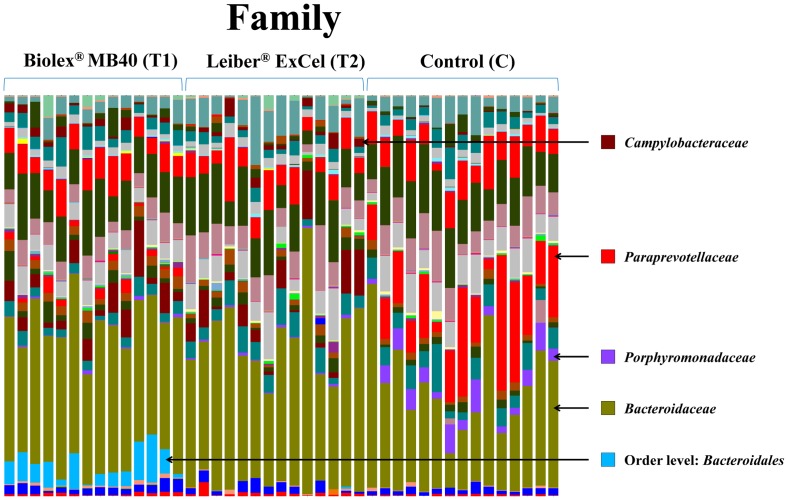
Overall cecal microbiota compositions of each sample with a family level. The 15 samples from left are T1 (normal feed with 0.2% Biolex^®^ MB40), 15 samples in the middle are T2 (normal feed with 0.2% Leiber^®^ ExCel) and next 15 samples are control (C; normal feed), respectively.

**Fig 6:**
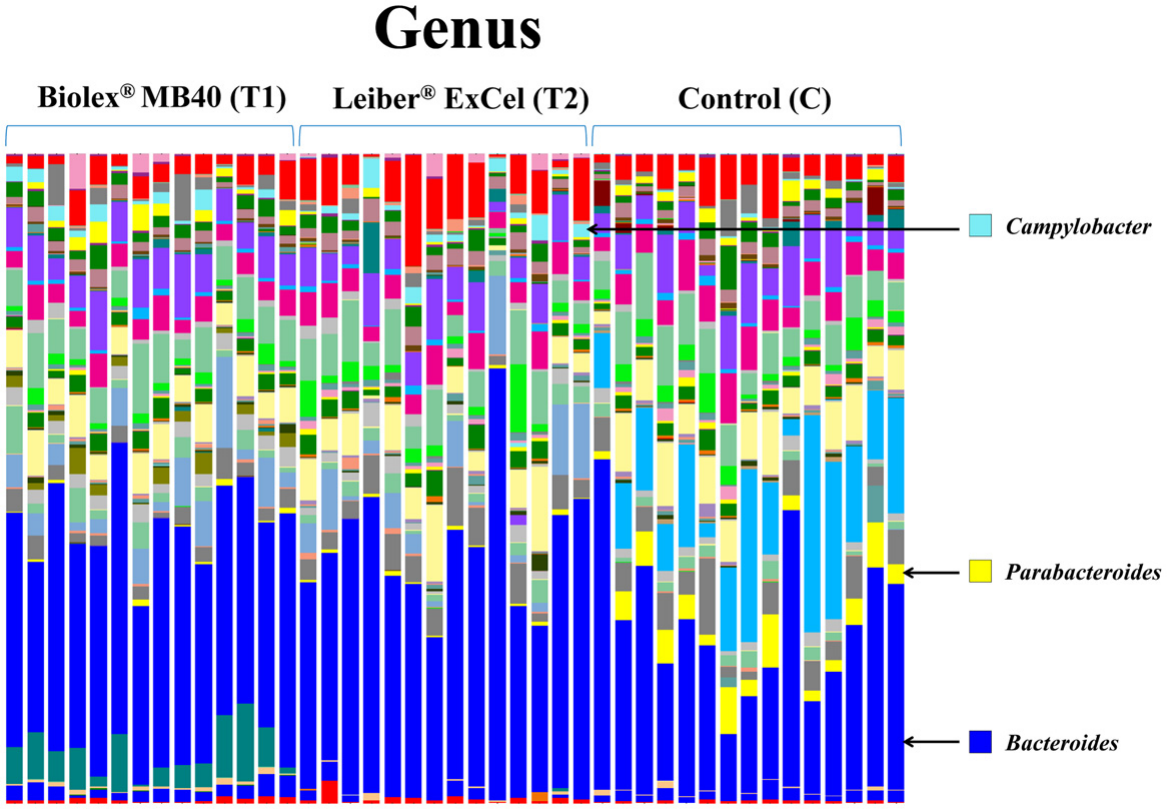
Overall cecal microbiota compositions of each sample with genus level. The 15 samples from left are T1 (normal feed with 0.2% Biolex^®^ MB40), 15 samples in the middle are T2 (normal feed with 0.2% Leiber^®^ ExCel) and next 15 samples are control (C; normal feed), respectively.

Figs 5 and 6 represent the relative distributions of OTUs in the cecal contents from each bird as a family and genus taxonomical level, respectively. As shown Fig 5, the control group exhibited distinct OTUs abundances at the family level compared to the other two treatment groups (T1: Biolex^®^ MB40 and T2: Leiber^®^ ExCel). Both families, *Paraprevotellaceae* and *Porphyromonadaceae*, were detected in all birds from the the control group with high amounts of OTUs and *Porphyromonadaceae* was further identified as genus *Parabacteroides* (Fig 5). The family *Campylobacteraceae* (Fig 5), genus *Campylobacter* (Fig 6), was present in all groups analyzed but the two treatment groups (T1: Biolex^®^ MB40 and T2: Leiber^®^ ExCel) exhibited significantly greater OTU abundance (Table 2). Interestingly, some bacteria from the order *Bacteroidales* OTUs (Fig 5) were detected only in the T1 (Biolex^®^ MB4) group but could not be further assigned as a particular family and genus level.

**Table 2:** Average of *Campylobacter* OTU abundance in a genus level among groups.

	Average ± SE* (%)		
Genus	Control (C)	Biolex MB40 (T1)	Leiber ExCel (T2)
*Campylobacter*	0.17 ± 0.03^b^	1.31 ± 0.27^a^	1.56 ± 0.04^a^
*Faecalibacterium*	0.52 ± 0.11^b^	0.86 ± 0.16^ab^	0.98 ± 0.21a^a^

SE*: Standard error

Lower case letters stand for significant differences among groups (*P* < 0.05).

### Effects of prebiotics on microbial populations

Although prebiotics based on yeast cell walls have been investigated their effects on gut microbial populations of chickens and applied to poultry industry in order to reduce foodborne pathogens, the exact mechanisms and functions of these prebiotics remain unclear and inconsistent results have been reported [51–53]. The MOS which is a main component of prebiotics used in this study has been known to not only enhance growth performance of birds [54] but also bind to the mannose-specific type-1 fimbriae receptor of pathogens to prevent colonization [55]. In addition, MOS may stimulate the growth of beneficial bacteria and enhance host immune responses [53]. In this study, there was a significant increase in genus *Faecalibacterium* OTUs, representing the phylum *Firmicutes* which are typically associated with health benefits as host commensal microorganisms [10] (Table 2).

### Detection of *Campylobacter*

*Campylobacter* is a commensal bacterium in poultry but one of the most important foodborne pathogens to humans originating from poultry and poultry products. Throughout sequencing analysis, we sorted out *Campylobacter* prevalence at the genus level to evaluate the effects of two commercial prebiotics and all 45 birds possessed *Campylobacter* with a range of 4.91 to 0.01%. As an average of *Campylobacter* OTU abundance, there was no difference between the two prebiotics treated groups (T1: 1.43% and T2: 1.78%), while control group (C: 0.17%) exhibited significantly lower *Campylobacter* abundance compared to the other groups (Table 2; Fig 5). In order to confirm and support *Campylobacter* prevalence based on the sequencing results, quantitative PCR (qPCR) was performed according to the previous report [56], Similar to sequencing data, *Campylobacter* DNA copy numbers as a log value in both T1 (3.85 ± 0.17) and T2 (3.71 ± 0.17) groups were greater than the control (3.34 ± 0.16) group. In the previous report using these same samples as the current study, Park et al. [21] concluded that the control group exhibited a significant *Campylobacter* increase compared to the other two treatment groups using a conventional cultural method based on Campy-Cefex selective media (BD Biosciences, San Jose, CA, USA). However, we could not compare *Campylobacter* genus percentage of the sequencing data with the respective *Campylobacter* colony forming units (CFU) on selective media since sequencing data represented only the relative abundance within the entire sequenced bacterial population and 16S rRNA genes can have multiple copies on genomic DNA. In addition, although Campy-Cefex agar media have been widely utilized for the *Campylobacter* quantitation, several studies have reported its limitations on the detection ability [57, 58]. Line and Berrang [58] reported that Campy-Cefex media exhibited less inhibitory effects on the background bacteria in chicken carcass rinsates and Chon et al. [58] also noted similar results demonstrating that Campy-Cefex media exhibits a low isolation rate, accuracy and selectivity in the enriched chicken carcass rinsates.

## Conclusions

Using high-throughput sequencing technologies, we were able to identify and delineate specific GIT microbiota patterns in long neck broilers fed yeast-based commercial prebiotics. We concluded that prebiotics additives derived from yeast cell walls altered cecal microbiota composition not only with changes in the phyla *Firmicutes* and *Proteobacteria* but also distinct changes in bacterial family and genus levels. Identifying specific microbial groups that tend to be predominant may help to better understand the interaction between host and GIT microbiota as well as microbiota changes when supplemented with food additives. Since the customized index primers utilized in this study for sequencing can generate a maximum of 384 different combinations, we could analyze over 300 samples in one reaction which offers advantages for larger studies utilizing more birds.

## Supporting Information

S1 Fig Taxonomic summary from phylum to genus levels. (PDF)

S1 Table Summary of reads number utilized in QIIME analyses. (XLSX)

S2 Table Relative OTUs abundance among samples. (XLSX)
